# Medaka (*Oryzias latipes*) *Dmrt3a* Is Involved in Male Fertility

**DOI:** 10.3390/ani14162406

**Published:** 2024-08-19

**Authors:** Ju Deng, Yan Huang, Jingjie Liang, Yuewen Jiang, Tiansheng Chen

**Affiliations:** 1 State Key Laboratory of Mariculture Breeding, Engineering Research Center of the Modern Technology for Eel Industry, Ministry of Education, Jimei University, Xiamen 361021, China; 202111908016@jmu.edu.cn (J.D.); 202161000121@jmu.edu.cn (Y.H.); 202261000039@jmu.edu.cn (J.L.); jyuewen@webmail.hzau.edu.cn (Y.J.); 2 Key Laboratory of Healthy Mariculture for the East China Sea, Ministry of Agriculture and Rural Affairs, Fisheries College, Jimei University, Xiamen 361021, China

**Keywords:** *Oryzias latipes*, *dmrt3a*, CRISPR/Cas9, spermatogenesis, mitochondria

## Abstract

**Simple Summary:**

*Dmrt3* plays pivotal roles in testicular development, but the precise molecular mechanisms remain unclear. In this study, we investigated the role of medaka (*Oryzias latipes*) *dmrt3* (*dmrt3a*) in testis development. This paper demonstrates that *dmrt3a* can maintain the number of germ cells and sperm motility, and it is expected to provide a theoretical basis for the diagnosis and treatment of human oligospermia and asthenospermia.

**Abstract:**

Research across various species has demonstrated that the doublesex and mab-3-related transcription factor 3 (*dmrt3*) plays pivotal roles in testis development. However, the precise molecular mechanisms of *dmrt3* remain unclear. In this study, we investigated the role of *dmrt3* (*dmrt3a*) in testis development using the model organism medaka (*Oryzias latipes*). SqRT-PCR and ISH analyses revealed that *dmrt3a* is predominantly expressed in the testis, especially in the spermatid and spermatozoon. Using CRISPR/Cas9, we generated two *dmrt3a* homozygous mutants (-8 bp and -11 bp), which exhibited significantly reduced fertilization rates and embryo production. Additionally, the number of germ cells and sperm motility were markedly decreased in the *dmrt3a* mutants, manifesting as the symptoms of asthenozoospermia and oligozoospermia. Interestingly, RNA-Seq analysis showed that the deficiency of *dmrt3a* could lead to a significant downregulation of numerous genes related to gonadal development and severe disruptions in mitochondrial function. These results suggested that *dmrt3a* is essential for spermatogenesis and spermatozoa energy production. This paper provides new insights and perspectives for further exploring the molecular mechanisms underlying spermatogenesis and addressing male reproductive issues.

## 1. Introduction

Reproduction is a critical life process for organisms, encompassing sex determination, sex differentiation, gonadal development, maturation, spawning, fertilization, and development. Gametogenesis serves as an essential component of sexual reproduction and ensures the continuation of reproduction and the evolution of life. The quantity and quality of gametes are crucial for successful animal breeding and for improving superior breeding stock. In recent years, a pivotal strategy in breeding programs and the enhancement of production traits has been the selection of male animals with exceptional sperm quality [1]. Sperm represent the most specialized and morphologically diverse group of specialized cells in sexually reproducing organisms, playing a crucial role in both the propagation and genetic diversity of animal species [2]. Spermatogenesis is an intricate and orderly process through which undifferentiated spermatogonia stem cells differentiate into haploid spermatozoa. This transformation involves a sequence of mitotic and meiotic divisions, encompassing key stages such as the proliferation and differentiation of spermatogonia, the meiotic division of spermatocytes, the maturation of round spermatids, and ultimately, the release of mature sperm into the lumen [3]. Numerous factors can impact the process of spermatogenesis including hormonal signals growth factors and other paracrine factors regulating germ cell development [4,5,6].

The *Dmrt* (doublesex and mad-3-related transcription factor) gene family is a crucial group of transcription factors that share a unique DNA binding motif known as the DM domain. This motif is commonly utilized in research related to sex determination and differentiation in vertebrates [7,8,9]. In fish, seven members of the *Dmrt* gene family have been identified, including *dmrt1*, *dmy*, *dmrt2a*, *dmrt2b*, *dmrt3a*, *dmrt4*, and *dmrt5* [10,11]. The expression of *dmrt1* has been extensively documented in male gonads across various species such as *Homo sapiens* [12,13], *Mus musculus* [14,15], *Gallus gallus* [16], *Oryzias latipes* [17], and *Xenopus laevis* [18,19]. This extensive documentation provides evidence of its role in the sex determination and differentiation of males.

Synteny analysis has revealed that *dmrt1*, *dmrt2* and *dmrt3* genes are conserved and closely linked throughout evolution [20,21], suggesting that these three genes may have evolved as closely related homologs through gene amplification. In certain species, such as humans, mice, and chickens, the *dmrt3* gene is found adjacent to the 3′ terminus of *dmrt1*. The expression of the *dmrt3* gene in gonadal development has been well documented across multiple species in recent years. Previous studies in mammals have suggested that the mutation of the *DMRT3* and *OAS3* complex could potentially result in gonadal disorders by affecting the expression levels of *ESR1* [22]. In the platypus, *Dmrt3* was found to be specifically involved in the adult testis rather than the ovary [23] while *Dmrt3*-deficient homozygous mice exhibited abnormal male sexual development [24]. In birds, *Dmrt3* was found to be highly expressed in males after gonadal differentiation [25].

Studies on fish species such as *Cynoglossus semilaevis* and *Takifugu rubripes* have shown a higher expression of the *dmrt3* gene in the testis compared to the ovary. Additionally, the expression pattern of *dmrt3a* in Japanese pufferfish gonads was similar to that of *dmrt1* [26], which has been proven to be related to the determination and development of testes in vertebrates [27,28]. Therefore, it can be concluded that *dmrt3* participates in the regulation of sex differentiation and male gonad development in vertebrates. Despite numerous studies on the expression patterns of the *dmrt3* gene, the underlying mechanisms remain largely unknown, particularly in fish. The medaka possesses a stable genetic XX/XY sex-determination mechanism similar to that of humans [29,30]. Additionally, it has a rapid reproductive rate, a short generation cycle, daily spawning, and transparent embryos. These characteristics make the medaka an excellent model organism for studying sex regulation and differentiation [31,32].

In this study, we investigated the role of *dmrt3a* in testis development in medaka by using CRISPR/Cas9 to generate *dmrt3a*-null medaka, uncovering the novel function of *dmrt3a* in spermatogenesis. Notably, the deletion of *dmrt3a* resulted in male infertility, characterized by a significant decrease in the germ cell number and a nearly complete loss of sperm motility. Our findings emphasize the crucial role of *dmrt3a* in both sperm production and energy supply. This paper provides valuable insights for addressing challenges related to low aquaculture efficiency and productivity.

## 2. Materials and Methods

### 2.1. Animals

Hd-rR-strain medaka were raised in a circulating aquaculture system at 28 °C with a light/dark cycle of 14:10 h [33,34]. Mature medaka were obtained through natural reproduction. Fish embryos were cultured in Medaka Embryo Medium (MEM) (1 g NaCl, 0.03 g KCl, 0.04 g CaCl_2_⋅2H_2_O, 0.163 g MgSO_4_⋅7H_2_O, and 1 mL 0.1% methylene blue in 1 L of ultrapure water, having adjusted pH to 7.0 with 1.25% NaHCO_3_) at 28 °C [33]. After hatching, the fry was nourished with freshly hatched brine shrimp two to three times daily and attained sexual maturity approximately within three months.

Medaka used in this study were approved by the Ethics Committee of Science and Technology of Jimei University (Approval Code: JMU202203009) and fish experiments were performed according to “Guide for the Care and Use of Laboratory Animals” [35].

### 2.2. Phylogenetic Tree Construction

We collected Dmrt3 amino acid sequences of different species including mammals, birds, reptiles, amphibians, and fish through NCBI and Ensembl databases. The specific information is shown in Table S1. MEGA7.0 (https://www.megasoftware.net/, accessed on 10 January 2023) with the neighbor-joining method was used to construct the phylogenetic tree. To verify the reliability of the evolutionary tree, the parameter of bootstrap replications was set to 1000.

### 2.3. Semi-Quantitative RT-PCR (SqRT-PCR)

To investigate the expression of *dmrt3a* in various tissues of adult medaka, 3 wild-type male and 3 wild-type female medaka that had reached sexual maturity (6 months) were euthanized by freezing on ice water for 3 min. Subsequently, tissues including the heart, liver, brain, eye, gill, muscle, kidney, testis, and ovary were carefully dissected and preserved in 1 mL of pre-cooled RNAiso Plus (9109, TaKaRa, Shanghai, China). The tissues were then ground in a Tissue Homogenizer (Tiss-24, Jingxin, Shanghai, China) for 3 min until they were completely homogenized. Total RNA was extracted from every tissue and reverse-transcribed into cDNA according to a previous study [36] and the PrimeScript^TM^ II 1st strand cDNA Synthesis Kit (6210A, TaKaRa, Beijing, China) was used for reverse-transcription-synthesized cDNA. SqRT-PCR was performed to analyze the *dmrt3a* mRNA relative expression among the ten tissues. The PCR programs were set as 95 °C for 3 min, followed by 33 cycles of 95 °C for 30 s, 58 °C for 30 s, and 72 °C for 5 min. *β-actin* of medaka was used as an internal reference and the primers for *dmrt3a* and *β-actin* are listed in Table S2. The DNA marker used in this experiment was purchased from Takara.

### 2.4. In Situ Hybridization (ISH)

The testes of adult medaka (6 months, *n* = 3) were fixed overnight in 4% paraform aldehyde (Servicebio, Wuhan, China) at room temperature to check the cellular localization of *dmrt3a* expression in testis by ISH. The tissues were embedded in paraffin and the sections of 5 μm thickness per slice were cut using microtome (Leica, Wetzlar, Germany). The DNA sequences used for antisense and sense mRNA probes were amplified using a set of primers (Table S2) and transcribed in vitro with Transcription Aid T7 High Yield Transcription Kit (AM1344, Thermo Fisher Scientific, Waltham, MA, USA). The mRNA probes were retrieved by Lithium chloride precipitation. Hybridization was performed with the RNA probes (5 ng/μL) diluted with hybridization solution at 65 °C for 12 to 16 h. RNA probes hybridized were detected using an anti-DIG AP antibody conjugated with alkaline phosphatase (A4955, Roche, Switzerland ) and signals were detected by BCIP (5-bromo-4-chloroindol-3-yl phosphate)/NBT (Nitro Blue Tetrazolium) system according to the manufacturer’s instructions (BCIP/NBT Chromogen Kit) (PR1100, Solarbio, Wuhan, China). The signals with *dmrt3a* antisense probes were observed and the images of ISH were taken under a Leica DMLA compound microscope (Leica, Buffalo Grove, IL, USA).

### 2.5. Generation of dmrt3a Mutants by CRISPR/Cas9

The *dmrt3* gene in medaka was named *dmrt3a* (Gene ID: 111947862, chromosome 9). The *dmrt3a*-knockout medaka were generated by CRISPR/Cas9 genome editing system-mediated mutagenesis according to our previous study [37,38]. Briefly, the guide ribonucleic acid (gRNA) 5′-ggggcgcgcggctgcgacacCGG-3′ was designed using the online CCTop website (https://cctop.cos.uni-heidelberg.de:8043/, accessed on 21 October 2021) against an optimal CRISPR site targeting exon 1 of *dmrt3a* (ENSORLG00000024387). The DNA sequence including medaka *dmrt3a* gRNA scaffold was amplified by ordinary PCR using Target-sgRNA-F and sgRNA common-R [39] (Table S2) and transcribed in vitro using Transcript Aid T7 High Yield Transcription Kit (K0441, Thermo Fisher Scientific, Waltham, MA, USA). A mixture containing transcribed sgRNA (50 ng/μL) and Cas9 mRNA (300 ng/μL) solution was microinjected into the fertilized one-cell-stage embryos [40]. The positive F0 medaka were backcrossed with the wild-type medaka to generate F1. F1 adult medaka with the same genotype (+/−) were intercrossed to generate F2 offspring, which contained wild-type (+ /+), heterozygote (+/−), and homozygote (−/−) genotypes.

Genomic DNA was extracted from some individuals (*n* = 10) injected at 4 days after fertilization to verify the presence of mutations and confirm the activity of the gRNA. PCR products covering the target site were amplified using PCR primers *dmrt3a*-JC-F and *dmrt3a*-JC-R (Table S2), which flank the site of deletion. These products were then inserted into the pMD-18T vector (6011, Takara, Beijing China) for sequencing the mutation type by Sanger sequencing. The sequences were further analyzed using SnapGene (https://www.snapgene.com/, accessed on 16 January 2023) and DNAMAN (https://www.lynnon.com/index.html, accessed on 3 April 2023). To conduct mutation screening, genomic DNA sequences were extracted by cutting the caudal fins of adult medaka using the established alkaline lysis method [34]. The embryos that were injected were raised to the F0 generation of adult fish. The positive F0 medaka were crossed with wild type to generate the F1 generation. F1 heterozygotes of the same genotypes can generate F2 mutants by self-crossing. Homozygous mutants in the F1 and F2 generations can be screened out using the method described above.

### 2.6. Analysis of Spawning and Fertility

To analyze the ability to reproduce offspring in mutants, homozygous mutant male fish (*n* = 5) reaching their maturity at 6 months were crossed with confirmed fertile WT females (*n* = 5) in a 1:1 female-to-male ratio. The medaka were separated by partitions the night before spawning. In the next morning, the partitions were removed to allow the parents to mix. We collected the embryos from the female fish after one hour, counted the number of ovulations by 5 pairs each for wild type, and calculated the number of mutants and fertility daily for a period of 20 days.

### 2.7. Hematoxylin–Eosin (H&E) Staining

For Hematoxylin and Eosin (H&E) staining, dissected testes (*n* = 3) were fixed in 4% fixative solution (Servicebio, Wuhan, China) for 48 h, washed in Phosphate Buffered Saline (PBS), dehydrated in methanol, then embedded in paraffin and sectioned. Then the deparaffinized slide (5 µm) was stained in H&E according to the standard process described in a previous study [41].

### 2.8. TUNEL Assay

We took out the gonad sections prepared in advance. TUNEL System kit (GDP1042, Servicebio, Wuhan, China) was used in this experiment according to the manufacturer’s protocol. Briefly, these tissues (*n* = 3) were paraffin-embedded for sectioning. After that, the specimens were subjected to conventional dewaxing to water by xylene and absolute alcohol. Proteinase K solution, diluted the reconstituted Proteinase K by diluting 1:500 in PBS (pH = 7.4), was put into incubating tissues and washed using PBS. Then, we carried out rupturing of membranes using triton, balancing the tissues by buffer and adding the TDT enzyme and dUTP to incubate. Meanwhile, DAPI solution was used to stain cell nucleus. Finally, the images were collected by a fluorescence microscope.

### 2.9. Sperm Motility Evaluation

Twelve sexually mature wild-type and *dmrt3a*^−^/^−^ male medaka of the same size and age were selected for sperm motility testing. The male medaka were separated from the females the day before the experiment began. Then, the fish were anesthetized with 0.1 g/mL of Ethly 3-aminobenzoate methanesulfonate in a 500 mL beaker filled with water. This was followed by dissection and removal of the spermathecae and transfer to the HBSS buffer (14025092, Thermo Fisher Scientific, USA). The sperm suspension was obtained by squeezing the testes using the tweezers. The related parameters of sperm activity were determined using Sperm Quality Analyzer system (BEION S6, Shanghai, China). This experiment was performed at room temperature. Briefly, 2.5 μL sperm suspension was combined with 2.5 μL distilled water quickly in a 200 μL PCR tube. Then, 5 μL of the mixture was dropped into a sperm counting chamber with a volume of 5 μL immediately and was placed quickly under the microscope. Clicking “Motion analysis” icon, the sperm motility parameters were captured and timed automatically. Sperm motility parameters were captured at 3 s intervals and data from 30 s after sperm activation were extracted for calculation. Sperm is immediately activated in water. Therefore, the time between sperm activation and capturing the first picture should be shortened as far as possible, controlling between 5 and 10 s. It is advisable for two people to operate simultaneously. We chose the following parameters to assess the sperm motility: progressive (PR), non-progressive (NP), and immotility (IM).

### 2.10. RNA Sequencing and Data Analysis

To explore the molecular mechanism of *dmrt3a* in testis development and gamete formation in medaka, we conducted High-Throughput Sequencing (RNA-Seq) on the testis in WT and *dmrt3a*^−/−^ adult medaka (*n* = 3), using the testis of the wild-type as the control group. The testes of three sexually mature WT and *dmrt3a*^−/−^ male medaka (6-month-old) were removed and total RNA was isolated using RNAiso Plus according to the manufacturer’s protocol, separately. The quality was detected using 1% agarose gels. Each RNA sample was divided into two. One was saved as backup for later experiments. Then, other samples were sent to Novogene Company (Beijing, China) for transcriptome sequencing using an Illumina Hiseq platform. By using NEBNext^®^ UltraTM RNA Library Prep Kit (E7770S, Beijing, China) 3 μg total RNA from each sample was used to generate sequencing libraries. The reference genome can be directly downloaded from the Ensemble database (https://useast.ensembl.org/index.html, accessed on 11 August 2023). The reads mapped to each gene was calculated by FeatureCounts v1.5.0-p3 (https://subread.sourceforge.net, accessed on 12 August 2023). After removing the unqualified Reads and filtering out the Clean Reads, the HISAT 2 2.2.1 (https://daehwankimlab.github.io/hisat2/download/, accessed on 18 August 2023) was used to accurately and quickly compare the Clean Reads with the reference genome to obtain the localization information of the Reads on the reference genome (GCA_002234675.1) and the Reads of the ribosomal RNAs that were compared to the reference genome were deleted in this process [42]. The correlation of gene expression levels between samples is an important indicator to test the reliability of an experiment and the reasonableness of sample selection. The closer the Pearson correlation coefficient between samples is to 1, the higher the similarity of expression patterns between samples will be. Differentially expressed genes (DEGs) with |log2 fold change| of more than 1, false discovery rate (FDR) of less than 0.05, and Fragments Per Kilobase of transcript per Million mapped reads (FPKM) values of more than 1 were defined as significantly differentially expressed genes. Moreover, GO (Gene Ontology) and KEGG (Kyoto Encyclopedia of Genes and Genomes) pathway analysis were carried out to classify these genes. The clusterProfiler_4.12.2 (https://www.bioconductor.org/packages/release/bioc/html/clusterProfiler.html, accessed on 30 August 2020) was used to analyze the GO functional enrichment of the differential gene sets and the KEGG pathway was analyzed with padj < 0.05 as the threshold for significant enrichment.

### 2.11. Quantitative Real-Time PCR (qRT-PCR)

To ascertain the accuracy of the transcriptome data, 6 upregulated and 6 downregulated differentially expressed genes from RNA-Seq date, including *ndufaf5*, *sdhaf3*, *hoga1*, *ndufb3*, *cenpk*, *optn*, *dazl*, *amh*, *cyp17a2*, *COX1*, *CYTB,* and *ND4*, were selected for qRT-PCR experiments with the primers (Table S3). The samples used for qRT-PCR were the same as those used for sequencing. The *β-actin* was used as an internal reference gene. The specific operations are as follows.

The spare RNA samples for transcriptome sequencing were reverse-transcribed into cDNA using the same method as in Section 2.3. Using Quantitative Real-time PCR Analyzer PCR Instrument (qTOWER3GIVD, Analytik Jena AG, Jena, Germany), qRT-PCR was carried out in 15 µL reaction volumes containing 1 µL of template cDNA (appropriate 30 ng), 5 µL 2 x ChamQ Universal SYBR qPCR Master Mix (Q71103AA, Vazyme, Nanjing, China), 1 µL of each primer (10 μM), and 8 µL sterilized distilled water. Reactions were performed in 96-well plates (Monad, Wuhan, China) at 95 °C for 2 min, followed by 40 cycles of 95 °C for 15 s, 60 °C for 20 s, and 60 °C for 15 s. Relative expression levels of the assayed genes were normalized by *β-actin* and calculated using the 2^−∆∆CT^ method. All data were expressed as means ± SEMs of at least three independent experiments.

### 2.12. Statistical Analysis

Microsoft Excel 2017 and GraphPad Prism 7.0 (San Diego, CA, USA, www.graphpad.com, accessed on 29 December 2023) were used for data statistics and analysis. qRT-PCR results were analyzed by using unpaired *t*-tests and the ovulation situation was carried out by one-way analysis of variance (ANOVA) followed by the Tukey test. Sperm motion analysis was performed by multiple unpaired *t*-tests and Student’s *t*-tests between two groups. Group results were expressed as means ± SEMs. Significant differences between groups were demonstrated by utilizing *: *p* < 0.05, **: *p* < 0.01, ***: *p* < 0.001, and ****: *p* < 0.0001.

## 3. Results

### 3.1. Genetic Structure and Phylogenetic Analysis of dmrt3a

The *dmrt3a* of medaka is located on chromosome 9, and the genomic DNA spans 3137 bp (Gene ID: 111947862), comprising two exons of 391 bp and 938 bp, encoding 442 amino acids (aa) (XP_023813900). It features a conserved DM DNA binding domain at 24–70 aa and a DMA structural domain at 229–271 aa (Figure S1A–D).

**Figure 1:**
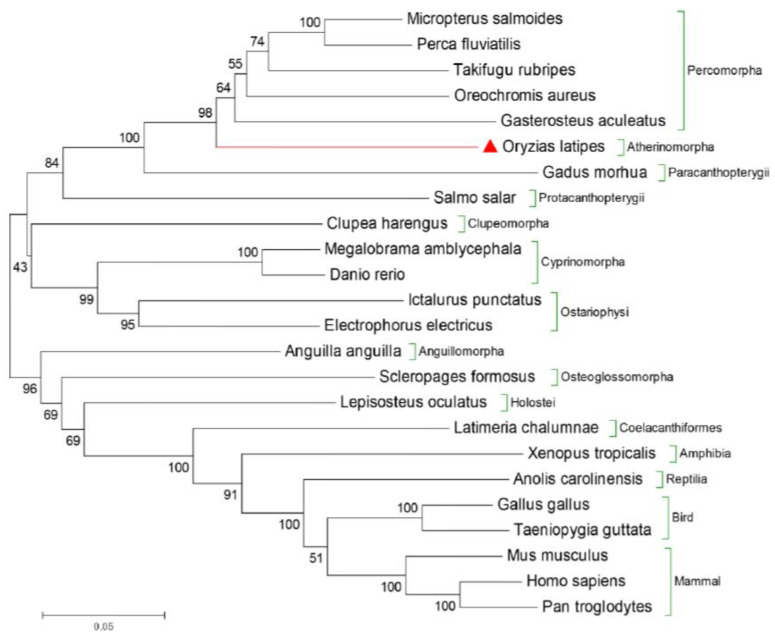
Phylogenetic reconstruction of Dmrt3 in vertebrates. The branching between medaka and others was deduced by using MEGA 7.0 software using Poisson correction distance based on the neighbor-joining method with 1000 bootstrap replicates. The numbers next to the branches represent bootstrap values. The medaka is marked by the red triangles. Brackets represent the orders of various species used. Green brackets represent different orders.

Phylogenetic analysis of *Dmrt3* across different vertebrates revealed that the phylogenetic tree was divided into two major branches. The *Dmrt3* genes from *Holostei*, *Coelacanthiformes*, Osteoglossomorpha, *Galliformes*, and higher vertebrates were clustered together in one large branch. However, the Clupeomorpha, *Atherinomorpha*, *Paracanthopterygii*, Ostariophysi, Clupeomorpha, and *Cyprinomorpha* were clustered into another large branch. Meanwhile, phylogenetic analysis showed that the medaka *dmrt3a* was closely related to Percomorpha (Figure 1).

### 3.2. Expression Patterns of dmrt3a

**Figure 2:**
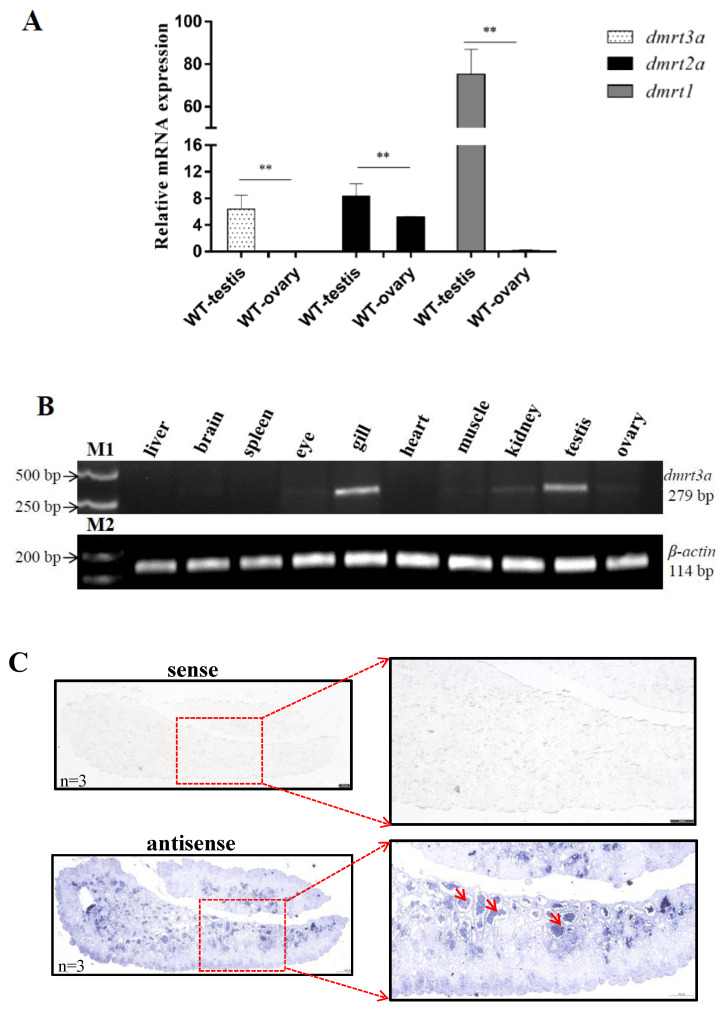
RNA expression of *dmrt3a*. (**A**) Transcriptome data analysis of medaka gonads from our laboratory. (**B**) SqRT-PCR was performed with cDNA from different tissues of adult medaka, with *β-actin* acting as an internal control. M1: DL 5000 DNA Marker. M2: DL 1000 DNA Marker. The lengths of the amplification products were 279 bp and 114 bp, respectively. (**C**) Expression of *dmrt3a* in adult testis by RNA in situ hybridization. The mature testis was hybridized with both sense and antisense RNA probes to detect the expression levels of *dmrt3a*. The details have been framed in red box and enlarged. The red arrow indicates the position of some *dmrt3a* in the testis. Scale bar = 100 µm. Original gels are presented in Supplementary Figure S2. **: *p* < 0.01.

Analysis of transcriptome data from our laboratory showed that the expression pattern of *dmrt3a* in medaka gonads is similar to that of *dmrt1*, with a significantly higher expression in the testis compared to the ovary, where it is nearly absent (Figure 2A). SqRT-PCR confirmed that *dmrt3a* is primarily expressed in the gill and testis, with minimal expression in the ovary (Figure 2B). In situ *hybridization* (ISH) further indicated that *dmrt3a* was specifically expressed in sperm cells (Figure 2C), suggesting a potential role in late-stage spermatogenesis and sperm motility.

### 3.3. Establishment of the dmrt3a Mutants

**Figure 3:**
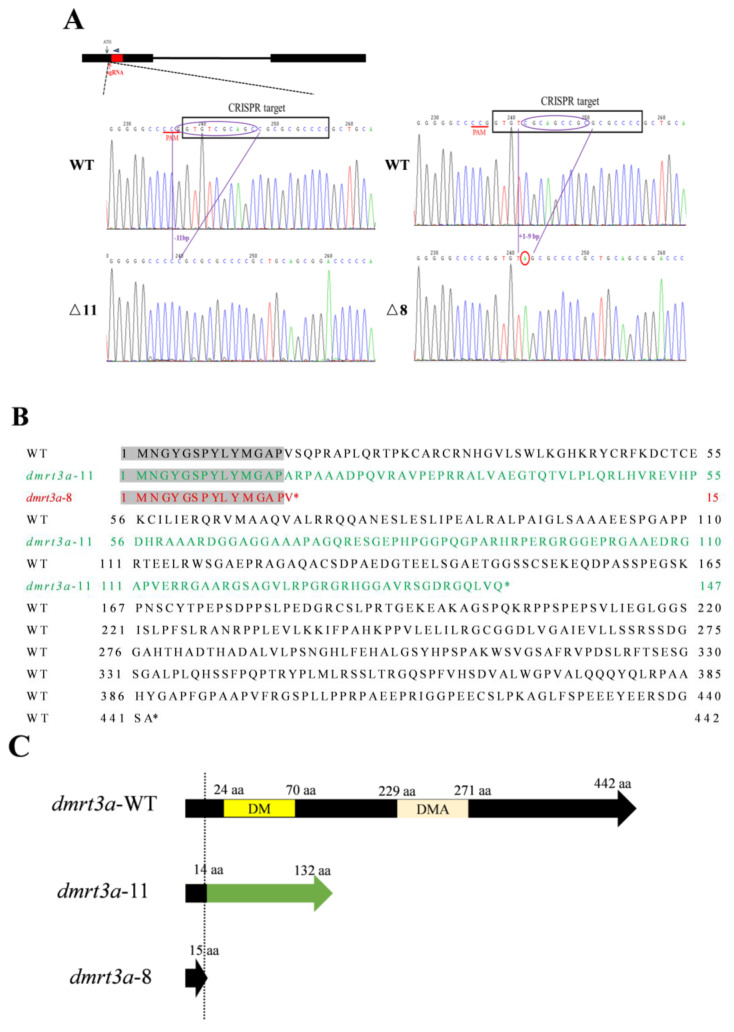
Generation of medaka *dmrt3a* mutants using CRISPR/Cas9 system. (**A**) Schematic representation of CRISPR/Cas9 target site and genotype of *dmrt3a* mutants. Two *dmrt3a*-specific mutations with 11 bp deletion and 8 bp deletion were generated in medaka. (**B**) The results of amino acid sequence alignment of *dmrt3a*-8^−/−^, *dmrt3a*-11^−/−^, and WT. The black letters represent the wild-type amino acid sequence. The green letters represent the amino acid sequence of the *dmrt3a*-11^−/−^. The red letters represent the amino acid sequence of the *dmrt3a*-8^−/−^. Gray represents the identical parts of the amino acid sequences of the three types of medaka. (**C**) The protein structures of WT and *dmrt3a*-8^−/−^ and *dmrt3a*-11^−/−^ mutations were predicted through Smart online website. The yellow part represents the DM domain. The light red part represents the DMA domain. The green part represents the sequence with a frameshift mutation in *dmrt3a*-11. WT: wild type. *dmrt3a*-8^−/−^ and *dmrt3a*-11^−/−^: different genotypes of mutants. *: stop codon.

Utilizing CRISPR/Cas9 technology, we knocked out the *dmrt3a* gene in medaka. Targeting the upstream of the conserved DM domain, we obtained two homozygous mutants, *dmrt3a*-8^−/−^ and *dmrt3a*-11^−/−^ (Figure 3A), which were confirmed by PCR and T7 Endonuclease I (Figure S2). Analysis showed that these mutants produce truncated Dmrt3a proteins of 147 and 15 amino acids, respectively, with both of them lacking the DM domain (Figure 3B,C), indicating a functional deficiency.

### 3.4. The Deletion of dmrt3a Leads to Male Reproductive Dysfunction

**Figure 4:**
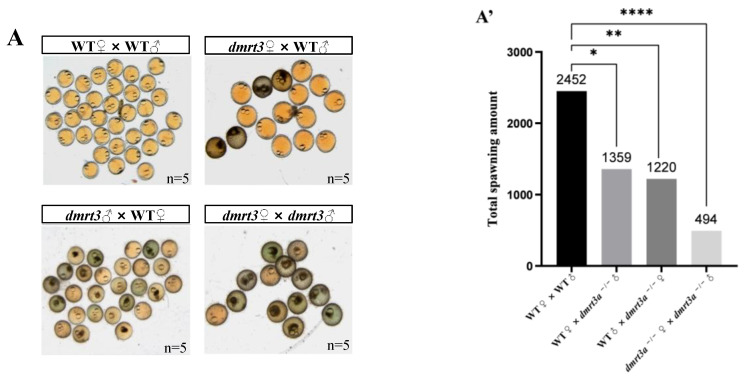

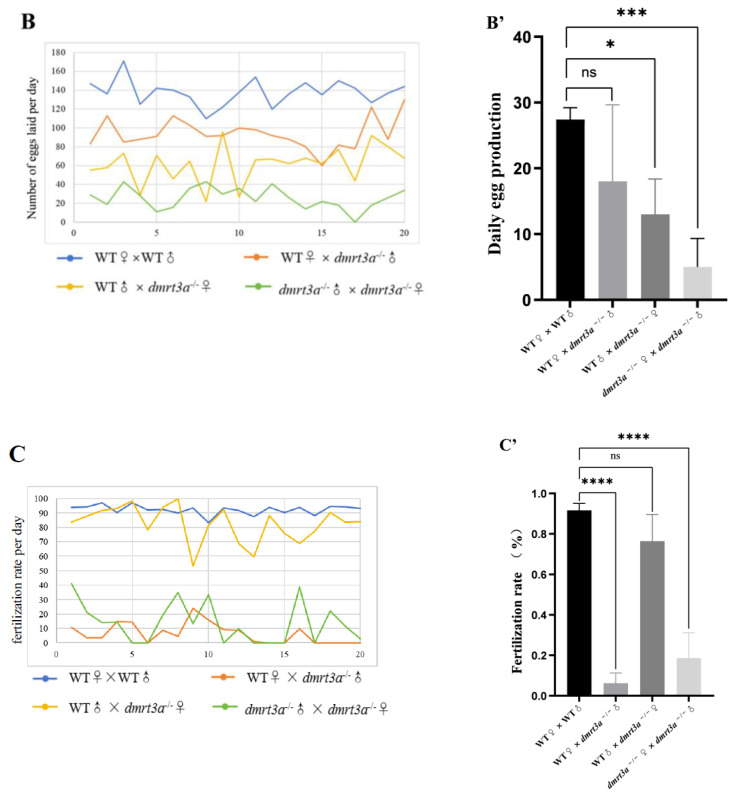
Comparison of fertility between WT and *dmrt3a*^−/−^ adult medaka. (**A**) Embryos of medaka intercrossed as WT♀× WT♂, WT♀ × *dmrt3a*^−/−^♂, WT♂ × *dmrt3a*^−/−^♀ and *dmrt3a*^−/−^♂ × *dmrt3a*^−/−^♀. (**A′**) The total numbers of eggs laid by the four groups of experimental fish over a 20-day period. (**B**) The line chart of the number of eggs laid in each group every day. (**B′**) The average ovulation numbers of different intercrossed adult medaka groups for 20 days. (**C**) The line chart of fertility rates every day in the five groups. (**C′**) Fertility rates of different intercrossed adult medaka groups for 20 days. All dates above are shown as means ± SDs and the experimental period was 20 days. Five balances were set for each group. ♂: male. ♀: female. “ns”: no significant difference. *: *p <* 0.05. **: *p* < 0.01, ***: *p* < 0.001. ****: *p* ≤ 0.0001. ns: no significance. Scale bar: 5 mm.

After obtaining the *dmrt3a* homozygous mutants, we observed consistent phenotypes in both mutants and noted lower fertilization rates in their offspring. Therefore, subsequent experimental studies uniformly used the *dmrt3a*-8 mutants, abbreviated as *dmrt3a*^−/−^. Five pairs of wild-type and *dmrt3a*^−/−^ medaka at the age of 6 months were chosen for breeding experiments. We separately counted 20 days of the spawning and fertilization status of the four groups WT♀× WT♂, WT♀ × *dmrt3a*^−/−^♂, WT♂ × *dmrt3a*^−/−^♀ and *dmrt3a*^−/−^♂ × *dmrt3a*^−/−^♀. We clearly observed that the quantity and quality of embryos including *dmrt3a*^−/−^♂ were extremely poor (Figure 4A,A′). By collecting embryos from each group, we found that the number of eggs laid per day was consistently at its lowest value in *dmrt3a*^−/−^♂ × *dmrt3a*^−/−^♀ during the 20-day experiment and the average daily eggs production was significantly different when comparing the *dmrt3a* mutant group to the WT group (Figure 4B,B′). By mating WT females with homozygous males, we found that although *dmrt3a*^−/−^♂ exhibited mating behavior, most of the embryos collected every day during mating were less fertilized (Figure 4C), with an average fertilization rate of 6% (*n* = 5, 1359 embryos accumulated) (Figure 4A′,C′). We also observed that mating homozygous mutant males with females resulted in a lower daily fertilization rate (Figure 4C), with an average fertilization rate of 18% (*n* = 5, 494 embryos accumulated) (Figure 4A′, C′). In contrast, WT males with females were able to successfully fertilize eggs at 92% (*n* = 5, 2452 embryos accumulated) (Figure 4A′,C′). These results indicate that the reproductive function of *dmrt3a*^−/−^ male fish is disrupted, and sperm motility is likely to be insufficient, leading to extremely low fertilization rates. 

### 3.5. dmrt3a Deficiency Leads to Medaka Sperm Dysfunction

**Figure 5:**
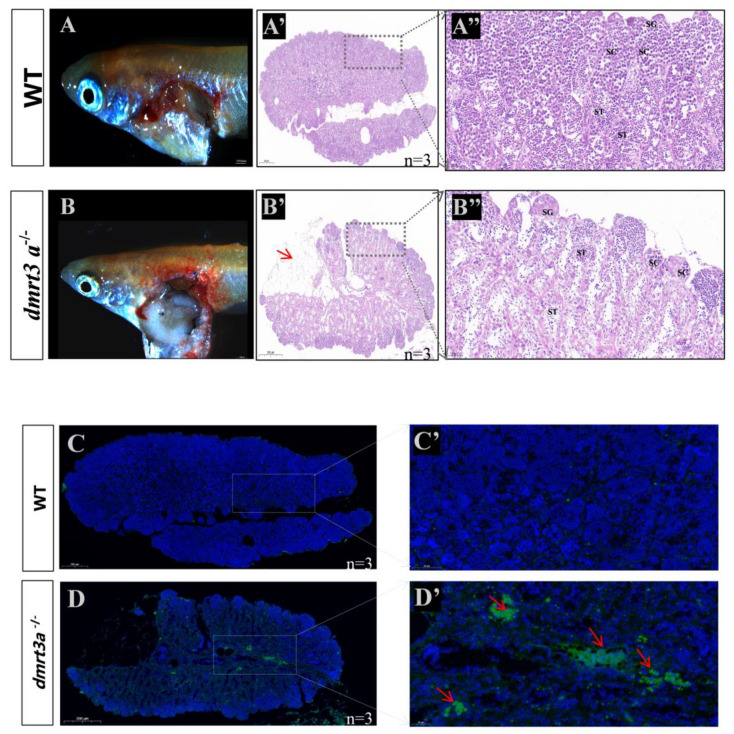

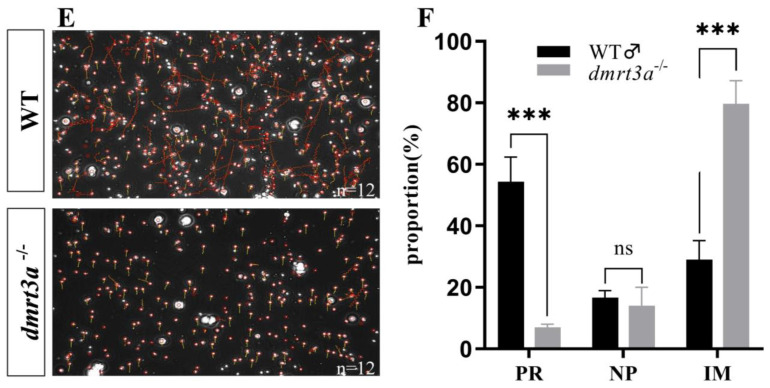
The internal structure of the testis and sperm motility in WT and *dmrt3a*^−/−^ medaka. (**A**,**B**) Appearances of WT and *dmrt3a*^−/−^ adult males and dissected testes. (**A′**,**B′**) H&E staining of testis from WT and *dmrt3a*^−/−^ medaka. Red arrow indicates the larger reticular vacuolar area in the testes (**A″**,**B″**) Higher magnification of testis section shown in (**A′**) and (**B′**). (**C**–**D′**) Apoptosis, detected using terminal deoxynucleotidyl transferase dUTP nick end labeling (TUNEL) in the WT and mutant testes. The red arrows indicate the details of the apoptotic cells. The green areas represent apoptotic signals. Note: SG: spermatogonia; SC: spermatocytes; ST: spermatid; SM: sperm. Scale bars: 1 mm in A and B; 200 μm in (**A′**,**B′**,**C**,**D**); 20 μm in (**A″**,**B″**); 50 μm in (**C′**,**D′**). (**E**) The sperm motility trajectories of WT and *dmrt3a* mutant medaka. The red lines represent the dynamic trajectory curve of normal sperm. The longer the line is, the farther it moves. The yellow lines represent the dynamic trajectory curve of abnormal sperm. (**F**) Comparison between WT and *dmrt3a* mutant sperm motility levels. PR: forward moving sperm. NP: non-forward-moving sperm. IM: immobile sperm. ns: no significance. ***: *p* < 0.001.

To further study the reason underlying reproductive dysfunction in *dmrt3a*^−/−^ males, we conducted a histological examination of the testes of WT and *dmrt3a* male mutants at the age of 6 months. Compared to the WT, no significant difference in appearance was observed in the *dmrt3a*^−/−^ testes (Figure 5A,B). However, mutants had larger reticular vacuolar tissue in the testes (Figure 5A′,B′) and a decrease in the number of mature sperm in the seminal vesicles (Figure 5A″,B″). TUNEL detection revealed a significant increase in the apoptosis rate of *dmrt3a*^−/−^ testicular somatic cells (Figure 5C,C′,D,D′). Additionally, the motility of sperm from two groups was analyzed using a sperm analyzer at 6 months of age. As shown in the sperm motility trajectory in Figure 5E, sperms of the *dmrt3a* mutant almost lost their motility compared with the WT. After multiple repetitions and statistical analyses, it was found that the *dmrt3a*^−/−^ mutant had 7% sperm with forward movement (PR), 14% sperm with non-forward movement (NP), and PR + NP = 21%, while the WT group had 54% sperm with forward movement (PR), 17% sperm with non-forward movement (NP), and 71% sperm with PR + NP (Figure 5F). According to the fourth edition of the WHO Laboratory Manual for Human Semen Examination and Treatment, the *dmrt3a* mutant male fish appeared asthenozoospermia. Furthermore, we also further collected the videos of spermatozoa from WT and mutant and found that sperm count and sperm motility were significantly lower in mutant compared to WT medaka (Videos S1,S2). 

### 3.6. Transcriptomic Analysis on dmrt3a-Knockout and Wild Type Medaka Testis

To explore the molecular mechanism of infertility resulting from *dmrt3a* knockout, we conducted transcriptomic analysis on wild-type and *dmrt3a* mutant fish of 6 mouths. Based on the FPKM values to indicate the gene expression levels, the correlation coefficient of the gene expression levels among samples exceeded 94%, which indicated that the reproducibility among the three samples was highly consistent and reliable (Figure S3A). The transcriptome analysis identified that 14,674 genes were expressed in both wild-type and *dmrt3a*^−/−^ homozygous testes, with 1150 genes specific to the *dmrt3a*^−/−^ testis and 906 genes specific to the wild-type testis (Figure S3B). There were 1645 upregulated genes and 1164 downregulated genes in the *dmrt3a^−/−^*, compared with the transcriptome of the wild type (Figure S3C).

**Figure 6:**
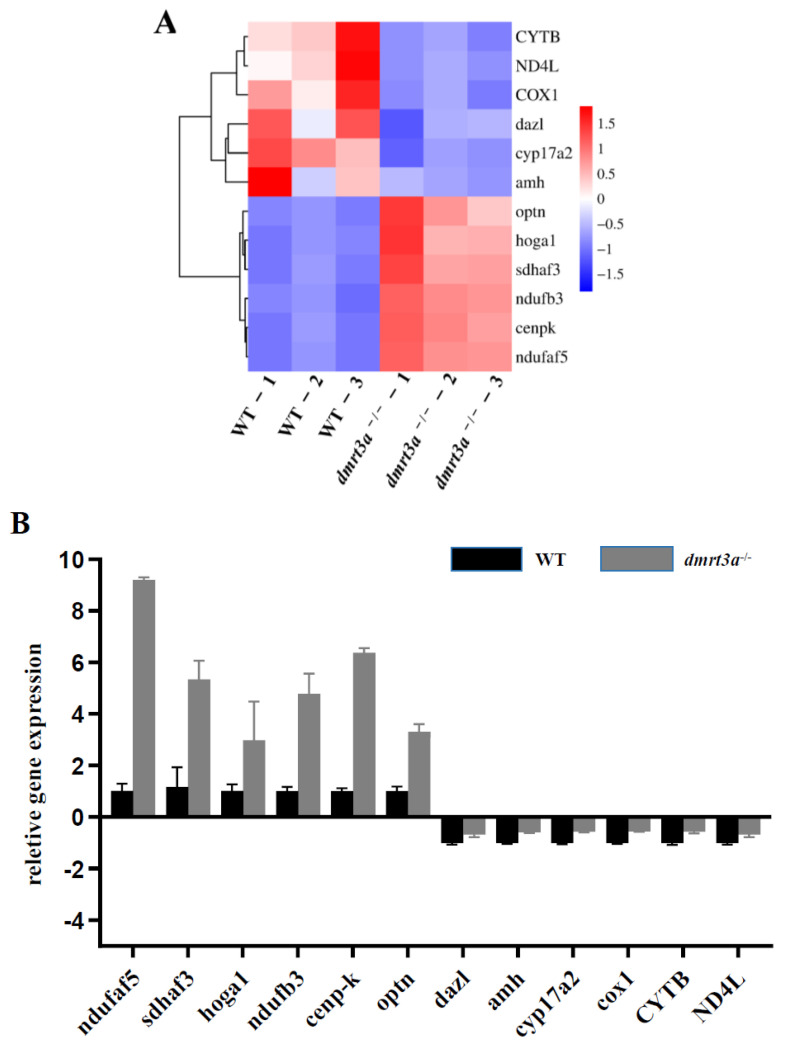
Validation of transcriptome data via qRT-PCR. (**A**) Clustering heatmap of genes selected to verify transcriptome data. Colors from red to blue represent the significant downregulation to upregulation. The expression levels of the same gene in different samples were normalized by using Z-score standardization. (**B**) Validation of transcriptome data by selecting six upregulated and six downregulated genes from the transcriptome of WT and (*dmrt3a*^−/−^) groups through qRT-PCR. The relative expression of genes between WT and *dmrt3a*^−/−^ medaka is shown as 2^−ΔΔCT^.

Subsequently, to validate the reliability of the transcriptome data, we performed qRT-PCR on 12 differentially expressed genes involved in mitochondrial respiratory chain, spermatogenesis, and germ cell maker genes in the transcriptome. The clustering heatmap of the 12 genes was generated by the relative RNA expression in WT and *dmrt3a*^−/−^ medaka from transcriptomic data (Figure 6A). The results indicated that the data obtained from the transcriptome were consistent with the results of qRT-PCR (Figure 6B). Therefore, transcriptome data are effective and reliable.

### 3.7. dmrt3a Deficiency Causes Testis Mitochondrial Dysfunction

**Figure 7:**
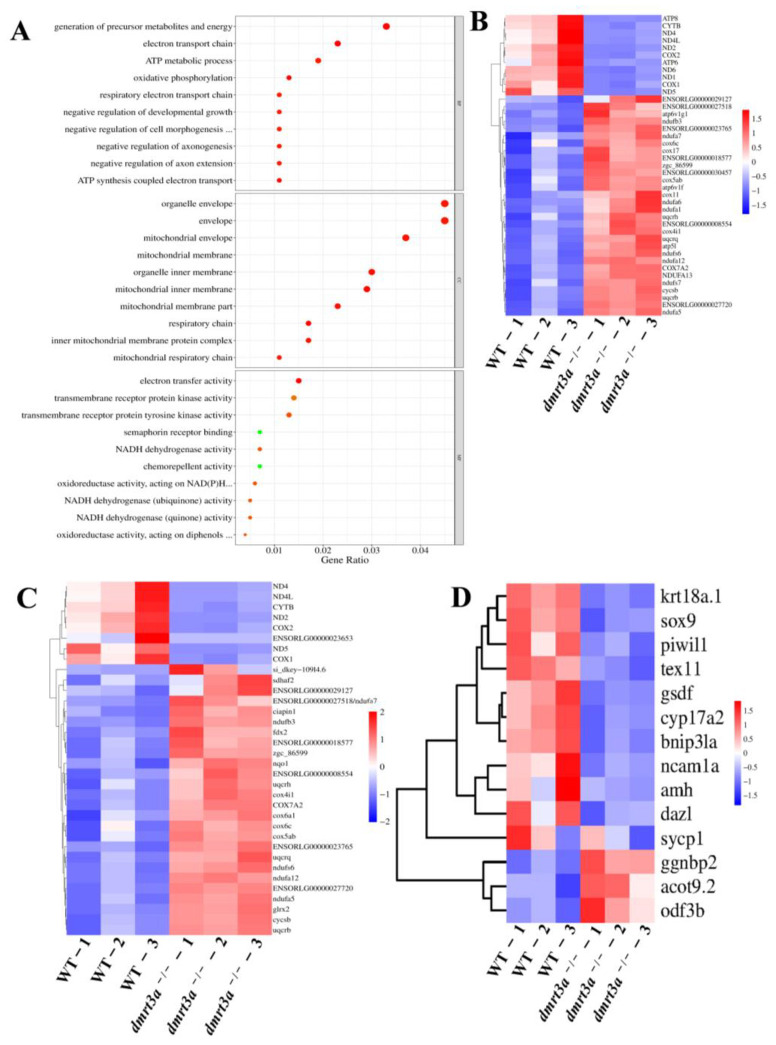
Bubble chart and clustering heatmaps of DEGs in differentially expressed genes in transcriptome data. (**A**) Bubble chart of the top 10 DEG-enriched GO pathways from BP, CC, and MF. The size of a dot represents the number of genes annotated to the GO pathway and the color (from red to green) represents the significance of the enrichment. (**B**) Oxidative phosphorylation pathway. (**C**) Electron transport chain pathway. (**D**) Clustering heatmaps of germ cell marker genes and spermatogenesis-related genes in the transcriptome data. Colors from red to blue represent the significant downregulation to upregulation. The expression levels of the same gene in different samples were normalized by using Z-score standardization.

To further trace the molecular mechanism of *dmrt3a*, we conducted GO and KEGG enrichment analysis on differentially expressed genes between the *dmrt3a* mutant and wild type. The results indicated that the top 30 pathways, including the molecular function (MF), biological process (BP), and cell component (CC) pathways, were primarily associated with the electron transport chain, oxidative phosphorylation, the generation of precursor metabolites, ATP synthesis, and other processes relevant to the structure and energy of mitochondria (Figure 7A). In addition, the top KEGG pathway was oxidative phosphorylation (Table S4). Clustering analysis showed the upregulated and downregulated genes in the pathways of oxidative phosphorylation and the electron transport chain (Figure 7B,C). We found that the genes of mitochondrial DNA (mtDNA) including *ND1*, *ND2*, *ND4*, *ND4L*, *ND5,* and *ND6*, which are related to the formation of energy transfer respiratory chains, were all significantly downregulated. Moreover, the genes associated with spermatogenesis, sperm quality, mitochondrial energy, and structure, including *COX1*, *COX2*, *CYTB*, *ATP6*, and *ATP8*, were significantly downregulated [43,44] (Table S5). Meanwhile, a significant change was observed in the expression of spermatogonia marker genes and androgen synthesis levels, including *dazl*, *piwil1*, and *cyp17a2* (Figure 7D; Table S6). Based on the aforementioned results, it can be inferred that the knockout of *dmrt3a* led to a significant disruption in the internal homeostasis of mitochondrial material synthesis and decomposition, particularly during spermatogenesis and sperm motility. The disruption was likely to impair sperm development and function, ultimately resulting in reduced fertility in male medaka.

## 4. Discussion

Research has demonstrated that *Dmrt3* is expressed in the testes of various species and plays a crucial role in sexual differentiation and development, including in humans [45], Japanese pufferfish [26], and zebrafish [46]. However, the underlying molecular mechanisms of *dmrt3* in testis development remain largely unclear. In this study, we utilized medaka as a model organism for reproductive and developmental regulation. We generated medaka *dmrt3a* mutants using CRISPR/Cas9 to investigate the role of *dmrt3a* in testis development. Subsequently, we comprehensively analyzed the phenotype of the *dmrt3a* mutants and investigated the molecular mechanisms involved in testis development, spermatogenesis, sperm maturation, sperm motility, and fertility to elucidate its function in male reproduction. This paper provides a crucial theoretical foundation and substantial genetic evidence for further understanding the molecular mechanism of *dmrt3* in testis development.

The *Dmrt* family genes play a significant role in the differentiation and development of gonads in animals [47]. As a member of the *Dmrt* family, *Dmrt3* has been well documented to play the important role in testis development [48,49]. Previous studies have demonstrated the expression pattern and impact of *Dmrt3* on the development of gonads in male individuals such as the mouse, *Micropterus salmoides,* and *Megalobrama amblycephala* [50,51]. Additionally, *dmrt3a* in medaka showed a peak of expression first detectable on day 4 during the early phase of embryogenesis and showed strong expression in adult testes and the differentiating gonads of medaka larvae [52]. Our experiment further supported these findings. Although studies have investigated the expression patterns of *dmrt3a* in testis development in medaka, they did not emphasize its function. In this study, the mutations of *dmrt3a* resulted in poor reproductive ability in male medaka. The embryos produced by the homozygous F2 generation exhibited significantly lower egg production and fertilization rates. However, the fertility of female medaka with *dmrt3a* mutation was not significantly affected. This phenomenon revealed that *dmrt3a* is indispensable for embryogenesis and the development of testes in medaka.

The deficiency of *dmrt3a* resulted in severe defects in the testes of adult medaka, with a high occurrence of vacuolation in numerous seminiferous tubules. Additionally, there was a significant downregulation of some key spermatogonia marker genes, such as *dazl* and *piwil1*, resulting in a notable reduction in the number of germ cells. Furthermore, it was found that the local cell apoptosis in the testis was the primary cause of significantly reduced germ cells. The transcriptome data revealed that several genes related to apoptosis, including *gadd45a*, *casp3*, *abraxas1*, *bcl2l1,* and *cycsb* (Table S6), were remarkably upregulated. Moreover, *Gadd45a* plays a crucial role in responding to DNA damage, and both DNA damage and apoptosis can induce the generation of *Gadd45a* [53,54]. Casp3 is a well-established effector of apoptosis, and its expression level can directly reflect the degree of cell apoptosis [55]. In addition to these findings from our study on medaka testis biology, we also observed a remarkable upregulation of the oxidative phosphorylation pathway. Excessive oxidative phosphorylation (OXPHOS) processes lead to reactive oxygen species (ROS) overproduction, which could cause elevated levels of ROS resulting in oxidative stress and disrupting the cellular function. Moreover, high levels of ROS can also lead to severe and disordered apoptotic reactions and DNA damage of germ cells, having adverse effects on sperm production and quality control mechanisms in the testis, further impacting the male reproduction and fertility [56,57]. However, more research is needed to fully understand the effects of all aspects of ROS on the male germ cells and reproductive function.

Sperm motility plays a crucial role in the process of male and female mating, enabling sperm to travel long distances through the female reproductive tract to facilitate fertilization [58]. Reduced sperm motility, known as asthenozoospermia, is a common cause of male infertility [59,60]. In *dmrt3a*^−/−^ medaka, there was a significant decrease in sperm movement speed or complete inactivity, with 84% of the sperm being immobile, indicating symptoms of asthenozoospermia. Additionally, this study found a severe disruption in sperm mitochondrial function. Previous research has shown that mitochondria are essential for maintaining sperm motility, capacitation, acrosome reaction, and DNA integrity through processes such as oxidative phosphorylation and the regulation of calcium ion homeostasis [61]. Sperm mitochondrial dysfunction could impair the generation of sufficient energy needed for sperm motility and may be an underlying cause of asthenozoospermia [62]. Mitochondrial dysfunction mainly includes mtDNA damage, abnormal levels of ROS (reactive oxygen species), changes in mitochondrial ultrastructure, etc. These problems can cause mitochondrial homeostasis imbalance, leading to impaired spermatozoa energy production.

Oxidative metabolism is crucial for the linear motility of sperm [63]. However, the excess by-products of OXPHOS, such as ROS, could be related to oxidative stress and damage sperm quality [64]. In the *dmrt3a*^−/−^ testis, the oxidative phosphorylation pathway was identified as the top-ranked pathway in transcriptome data analysis based on the KEGG database. This could lead to the overproduction of ROS, resulting in mtDNA damage and ultimately reducing sperm motility. Additionally, studies have shown that when ROS production exceeds the limited antioxidant defenses of sperm, mitochondrial DNA becomes susceptible to ROS damage [65]. This can disrupt protein synthesis encoded by mitochondrial DNA, harm the sperm plasma membrane, and potentially lead to various pathophysiological outcomes such as aging and apoptosis in vitro. These effects can further impact mitochondrial function and potentially result in infertility [66], which aligns with the reduced sperm motility phenotype observed in *dmrt3a* mutants. Therefore, our findings suggest that excessive ROS production induces structural damage to mitochondria, disrupting the overall energy supply chain and impairing sperm motility. This cascade of events ultimately contributes to male infertility.

Meanwhile, genes from mtDNAs, including NADH dehydrogenase (complex I), coenzyme Q—cytochrome c reductase/cytochrome b (complex III), cytochrome c oxidase (complex IV), and ATP synthase, were significantly reduced (Table S5). It has been demonstrated that mtDNA defects are strongly associated with male infertility [67]. Furthermore, an increase in the total mtDNA copy number in mtDNA mutator mice has been shown to reduce mitochondrial aberrations in spermatocytes and spermatids within the testis [68]. Organisms may suffer from mitochondrial DNA diseases as a result of mtDNA mutation or deletion [69]. Therefore, our study suggests that decreased mtDNA levels could induce mitochondrial dysfunction in medaka. Additionally, we observed significant disruptions in the mitochondrial structure and energy synthesis pathway, specifically affecting components such as the electron transport chain, mitochondrial membrane integrity, and NADH dehydrogenase activity. These disruptions led to imbalances in the synthesis, decomposition, and transport homeostasis of various substances within the testis, further resulting in inadequate energy supply and impaired sperm transportation. Consequently, it is crucial to protect and maintain sperm mitochondrial function for maintaining normal reproductive function.

## 5. Conclusions

In summary, our study investigated the function and molecular mechanism of medaka *dmrt3a* with *dmrt3a* mutants generated by the CRISPR/Cas9 system. The deficiency of *dmrt3a* could lead to a significant decrease in the number of germ cells and sperm motility, which is strongly associated with the imbalance of mitochondrial homeostasis in mutant testes. The results not only indicated that *dmrt3a* is necessary for maintaining sperm count and motility but also provided insights for the study of oligozoospermia and asthenozoospermia in humans.

## Data Availability

The transcriptome data (BioProject accession number, PRJNA1098984) were submitted to the NCBI SRA database and the datasets used or analyzed during the current study are available from the corresponding author upon reasonable request.

## Supplementary Materials

The following supporting information can be downloaded at: https://www.mdpi.com/article/10.3390/ani14162406/s1, Figure S1: Information on *dmrt3a* ORF and structure; Figure S2: The process of screening and identifying mutants using PCR and T7 Endonuclease I; Figure S3: Comparative transcriptome analysis of WT and *dmrt3a*^−/−^; Table S1: Amino acid sequence information for constructing Dmrt3 phylogenetic tree; Table S2: Primer sequences and usage list used in PCR reactions; Table S3: Primer sequences used in qRT-PCR analyses; Table S4: The top 10 DEG-enriched KEGG pathways; Table S5: The expression of mitochondrial protein-coding genes in medaka testis; Table S6: Changes in the expression genes; Video S1: The video of sperm motility of *dmrt3a*^−/−^; Video S2: The video of sperm motility of WT.
