# Supplementary material for: Medaka (Oryzias latipes) Dmrt3a Is Involved in Male Fertility
Source: Animals (Basel). 2024 Aug 19;14(16):2406. doi: 10.3390/ani14162406 (PMC11350882; doi:10.3390/ani14162406)
Supplement: Supplementary file 1 [file animals-14-02406-s001.zip › animals-3143447-supplementary/animals-3143447-supplementary/Supplemental- files- Tables and figures-dmrt3/Supplemental Tables-dmrt3.pdf]

Supplemental Tables

Supplemental Table S1 - Amino acid sequence information for constructing Dmrt3 phylogenetic tree

Superorder/order	species	accession No.
	Homo sapiens	NP_067063.1
Mammal	Mus musculus	NP_796334.2
	Pan troglodytes	XP_054513866.1
Bird	Gallus gallus	XP_429193.2
	Taeniopygia guttata	XP_030114282.1
Reptilia	Anolis carolinensis	XP_003216535.1
Amphibia	Xenopus tropicalis	NP_001243149.1
Teleost		
Cyprinomorpha	Megalobrama amblycephala	AJD87235.1
	Danio rerio	NP_001005779.2
Atherinomorpha	Oryzias latipes	XP_023813900.1
Protacanthopterygii	Salmo salar	XP_013999084.2
Paracanthopterygii	Gadus morhua	XP_030215428.1
Tetraodontiformes	Takifugu rubripes	NP_001033034.1
Ostariophysi	Ictalurus punctatus	XP_017308040.1
Clupeomorpha	Clupea harengus	XP_012684056.2
Anguillomorpha	Electrophorus electricus	XP_026884873.1
	Anguilla anguilla	XP_035235688.1
Osteoglossomorpha	Scleropages formosus	XP_029115443.1
Holostei	Lepisosteus oculatus	XP_015194662.1
Coelacanthiformes	Latimeria chalumnae	XP_006001258.1
	Micropterus salmoides	UBF41578.1
	Takifugu rubripes	NP_001033034.1
Percomorpha	Gasterosteus aculeatus	ENSGACP00000019142
	Perca fluviatilis	XP_039660421.1
	Oreochromis aureus	XP_031612438.1

Supplemental Table S2 - Primers sequences and usage list used in PCR reactions

Primer Names	Sequence (5' to 3')	Exon location	Accession number	Size (bp)
**RT-PCR**				
dmrt3a-RT-PCR-F	CGCCAGCAGGCCAACGAGAGTC	1	NC_019867	279
dmrt3a-RT-PCR-R	GTTCGGTTTGGAGCCTTCGGGG	2		
**ISH probe**				
dmrt3a-probe-F	TAATACGACTCACTATAGGGGCTC TCCGTACCTCTACATGG	1	NC_019867	409
dmrt3a-probe-R	CAGAGCCTCACCTGAGCACG	1		
dmrt3a-antiprobe-F	GCTCTCCGTACCTCTACATGG	1	NC_019867	409
dmrt3a-antiprobe-R	TAATACGACTCACTATAGGGCAGA GCCTCACCTGAGCACG	1		
**PCR amplification for the DNA template of sgRNAs**				
Target-sgRNA-F	TAATACGACTCACTATAGGGGGGG CGCGCGGCTGCGACACGTTTTAG AGCTAGAAATAGC	1	-	120
sgRNA Common-R	AAAAGCACCGACTCGGTGCC	1		
**Detection of *dmrt3a* knockout RT-PCR**				
dmrt3a-JC-F	GTGACCACGGGGGACCTTTGC	0	NC_019867	386
dmrt3a-JC-R	GCCGCTCGATGATCAGGATGC	1		
**RT-PCR/qRT-PCR**				
β-actin-F	TATCATTCGCCTGAAACCGAT	1	NM_001104808.1	114
β-actin-R	CTTTGCACATGCCAGATCCG	2		

Supplemental Table S3 – Primer sequences used in qRT-PCR analyses

Primer Names	Accession number	Sequence (5' to 3')	Exon location	Size (bp)
ndufaf5-qPCR-F	XM_023953723.1	TGACGGATCTGCAAGGGATG	8	173
ndufaf5-qPCR-R		GTGAGGCTTCCAGCCAATCA	10	
sdhaf3-qPCR-F	XM_023960049.1	ATGGCAGAGTGGGAGAACTACA	1	203
sdhaf3-qPCR-R		TTGTCCTCGCGGATGTCGAA	2	
hoga1-qPCR-F	XM_023958047	CTCGGATCGGCCTGATTGTT	6	242
hoga1-qPCR-R		CAACTTTCTGGTCACGGCGG	8	
ndufb3-qPCR-F	XM_004079017.4	ACAAAAGGAACACCGCTGGA	2	192
ndufb3-qPCR-R		AGCGTACTCTATTGCCAGAGC	3	
cenpk-qPCR-F	XM_004066183.3	ACCACACTTCAGTGACAGCC	3	243
cenpk-qPCR-R		TCCAGCCATTTCTGTTCTAGCTC	8	
optn-qPCR-F	XM_004086957.4	TCGGATTTCTATGCCGAGCG	11	226
optn-qPCR-R		GCTGCCAGTCGGTACTTCT	13	
dazl-qPCR-F	XM_020706872.2	GGGCTCTGCAAAGGATACGG	3	180
dazl-qPCR-R		GGGATTCATCCAGGCACCAT	6	
amh-qPCR-F	NM_001360941.1	CAGATGGGCAGTGGTACAGG	1	283
amh-qPCR-R		AGCAACTTCCCATCAGGCTC	3	
cyp17a2-qPCR-F	XM_023965771.1	GCCTCCCGCCTCACCTCTTCA	1	256
cyp17a2-qPCR-R		GTCGGCTTCCTTCCCCAAACA	3	
COX1-qPCR-F	NC_004387.1	AGCCCACCACATATTCACCG	1	300
COX1-qPCR-R		GCAAATACGGCCCCCATAGA	1	
CYTB-qPCR-F	NC_004387.1	CCTTCACATTGGACGAGGCT	1	207
CYTB-qPCR-R		ATTCATTGGACGAGGGCGTT	1	
ND4L-qPCR-F	NC_004387.1	CGAAAGCACCTCTTATCCGC	1	204
ND4L-qPCR-R		TAAACGATCAGTGCCGTGGG	1	
β-actin-F	NM_001104808.1	TATCATTCGCCTGAAACCGAT	1	114
β-actin-R		CTTTGCACATGCCAGATCCG	2	

Supplemental Table S4 – The top 10 DEGs enriched KEGG pathways.

KEGGID	Description	GeneRatio	pvalue
ola00190	Oxidative phosphorylation	42/758	1.61E-10
ola04260	Cardiac muscle contraction	33/758	4.86E-06
ola04512	ECM-receptor interaction	25/758	0.001081703
ola00630	Glyoxylate and dicarboxylate metabolism	10/758	0.003726065
ola03040	Spliceosome	25/758	0.005070062
ola03013	Nucleocytoplasmic transport	21/758	0.005636209
ola04510	Focal adhesion	45/758	0.005858694
ola03440	Homologous recombination	10/758	0.006923724
ola00130	Ubiquinone and other terpenoid-quinone biosynthesis	5/758	0.012531058
ola00650	Butanoate metabolism	6/758	0.02020542

Supplemental Table S5 – The expression of mitochondrial protein-coding genes [1] in medaka testis.

mtDNA protein-coding genes						
**Category**	**Ensemble ID**	**Gene name**	**FPKM (WT)**	**FPKM (MT)**	**log2FoldChange**	**pvalue**
NADH Dehydrogenase (complex I)	ENSORLG00000021753	MT-*ND1*	2527.225567	700.2918417	-1.851638227	1.40E-16
	ENSORLG00000021757	MT-*ND2*	6855.754655	2330.697252	-1.556611052	4.09E-13
	ENSORLG00000021775	MT-*ND4*	23282.90863	6610.65124	-1.816420537	1.06E-11
	ENSORLG00000021774	MT-*ND4L*	2893.03834	683.3065953	-2.082107148	2.64E-12
	ENSORLG00000021779	MT-*ND5*	13486.16683	2598.915862	-2.375487023	1.64E-24
	ENSORLG00000021780	MT-*ND6*	3142.951108	926.3988023	-1.762516285	3.79E-16
Coenzyme Q - cytochrome c reductase/Cytochrome b (complex III)	ENSORLG00000021782	MT-*CYTB*	55977.71089	15638.81109	-1.839728648	6.37E-13
Cytochrome c oxidase (complex IV)	ENSORLG00000021763	MT-*COX1*	152643.4946	74682.12884	-1.031331558	2.18E-07
	ENSORLG00000021766	MT-*COX2*	44273.01793	19698.18249	-1.168373919	5.36E-08
	ENSORLG00000021770	MT-*COX3*	40290.53683	29294.60993	-0.459811124	0.025772293
ATP synthase	ENSORLG00000021769	MT-*ATP6*	18109.24444	6842.476057	-1.404151273	2.53E-08
	ENSORLG00000021768	MT-*ATP8*	149.3214763	32.06897681	-2.219618176	2.33E-09

FPKM: Fragments Per Kilobase of transcript per Million mapped reads.

Supplemental Table S6 – Changes in the expression genes.

Gene name	Ensemble ID	FPKM (WT)	FPKM (MT)	log2FoldChange	pvalue
**Spermatogonia marker genes**					
dazl	ENSORLG00000004848	4481.919323	3036.369637	-0.561703529	0.002540892
piwil1	ENSORLG00000013695	26395.82406	18970.12291	-0.476574217	0.001653465
**Apoptosis**					
gadd45a	ENSORLG00000005244	219.144061	653.4486992	1.575631647	0.0000000000000273
abraxas1	ENSORLG00000022037	147.4889324	462.1858373	1.646040284	0.000000000219
bcl2l1	ENSORLG00000025151	985.5205326	3039.434883	1.624413639	0.0000000195
bcl2l10	ENSORLG00000012758	1901.625923	3192.710488	0.747407894	0.001371469
bcl2l13	ENSORLG00000015896	1923.44617	2559.176563	0.41190982	0.014350433
cycsb	ENSORLG00000027899	929.6743992	2057.725456	1.145714682	0.000000046
casp3	ENSORLG00000008601	93.30176148	164.3426408	0.814239871	0.033214119
casp2	ENSORLG00000012073	1525.014441	2140.355197	0.488743604	0.005399623
aven	ENSORLG00000029866	119.1120323	239.8742159	1.010667316	0.000636853
CAAP1	ENSORLG00000004231	279.4507195	405.5177811	0.536153944	0.009254229
gadd45gip1	ENSORLG00000016597	148.0567079	226.9347734	0.615207014	0.008179682
cenpk	ENSORLG00000012705	2682.681196	872.3240225	1.620346819	2.59E-18
**Androgens**					
hsd17b3	ENSORLG00000004277	662.7790245	388.9751394	-0.768916378	0.029168687
cyp17a2	ENSORLG00000002242	2261.797678	1253.090437	-0.851902102	0.000000758
ar-alpha	ENSORLG00000008220	750.1577137	486.7557884	-0.623715883	0.001921847
ar	ENSORLG00000009520	155.3377566	81.35180843	-0.931761584	0.002873292

FPKM: Fragments Per Kilobase of transcript per Million mapped reads.

1. Jiang M, Kauppila T E S, Motori E, et al. Increased Total mtDNA Copy Number Cures Male Infertility Despite Unaltered mtDNA Mutation Load. *Cell Metabolism*. **2017**, 26, 429-436, https://doi.org/10.1016/j.cmet.2017.07.003.
