# Supplementary material for: Medaka (Oryzias latipes) Dmrt3a Is Involved in Male Fertility
Source: Animals (Basel). 2024 Aug 19;14(16):2406. doi: 10.3390/ani14162406 (PMC11350882; doi:10.3390/ani14162406)

Supplementary Figures

Supplementary Figure S1 – The information of *dmrt3a* ORF and structure. (A) Chromosome 9 of Japanese medaka. (B) *dmrt3a* gene structure. E1 and E2 indicate exons. (C) Protein structure of *dmrt3a*. yellow and grey areas show DM and DMA domains. (D) The OFR sequence, amino acid sequence and the major functional domains of medaka *a*. The yellow and gray shaded areas are the DM and DMA structures sequence, respectively, with the initiation codon ATG and the termination codon TGA bolded.

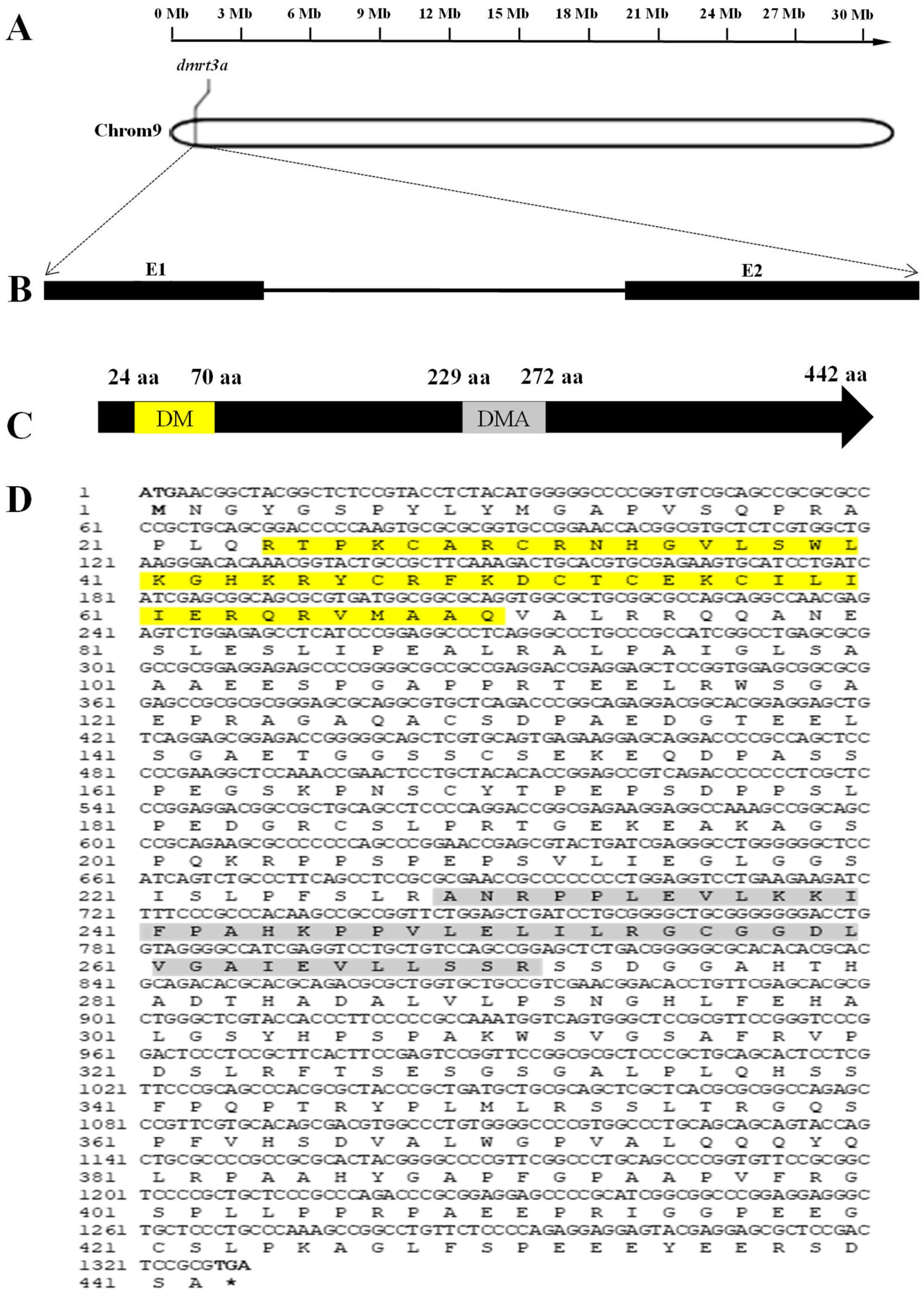

Supplementary Figure S2 – The process of screening and identifying mutants using PCR and T7 Endonuclease I. M1: DL 5000 DNA Maker; M2: DL 1000 DNA Maker; WT: wildtype medaka of positive control; The numbers marked in red represent the PCR samples to be mixed with WT for the second T7E1 test. The lengths of the amplification products were 386 bp. The amplification primers are indicated by black arrows, and the target sample is represented by the red box.

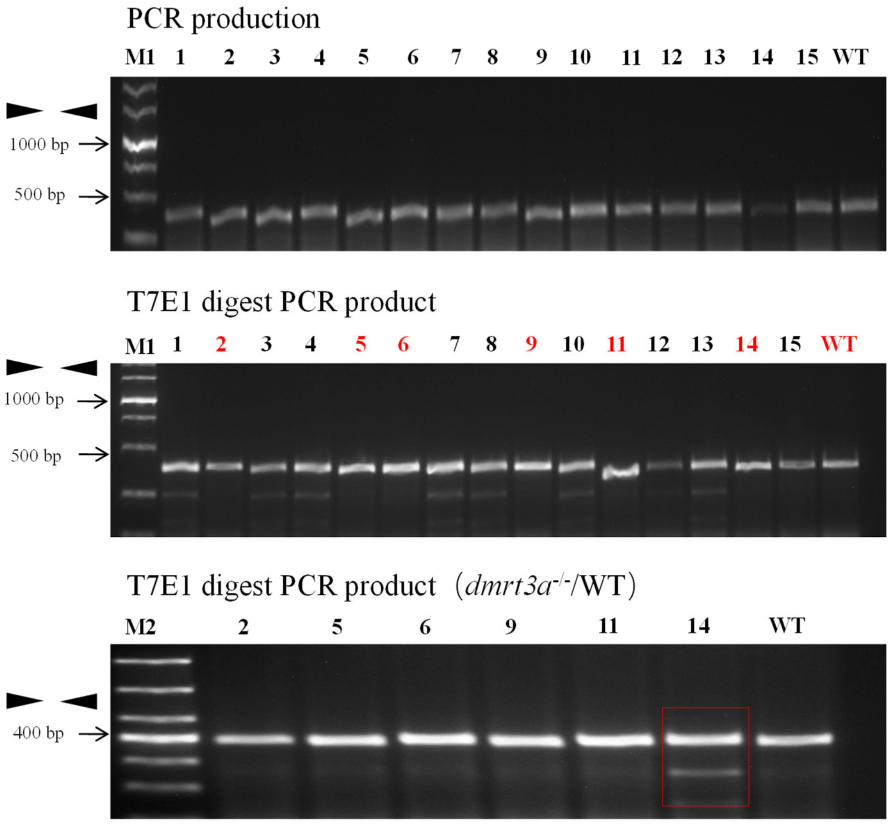

Supplementary Figure S3 – Comparative transcriptome analysis between WT and *dmrt3a*$^{-/-}$. (A) Heatmap shows the Pearson correlations among WT and *dmrt3a*$^{-/-}$. (B) The Venn diagram illustrates the number of expressed genes in mutants, WT samples, and genes that are co-expressed. (C) Volcano map shows the number of upregulated and downregulated genes in transcriptome.

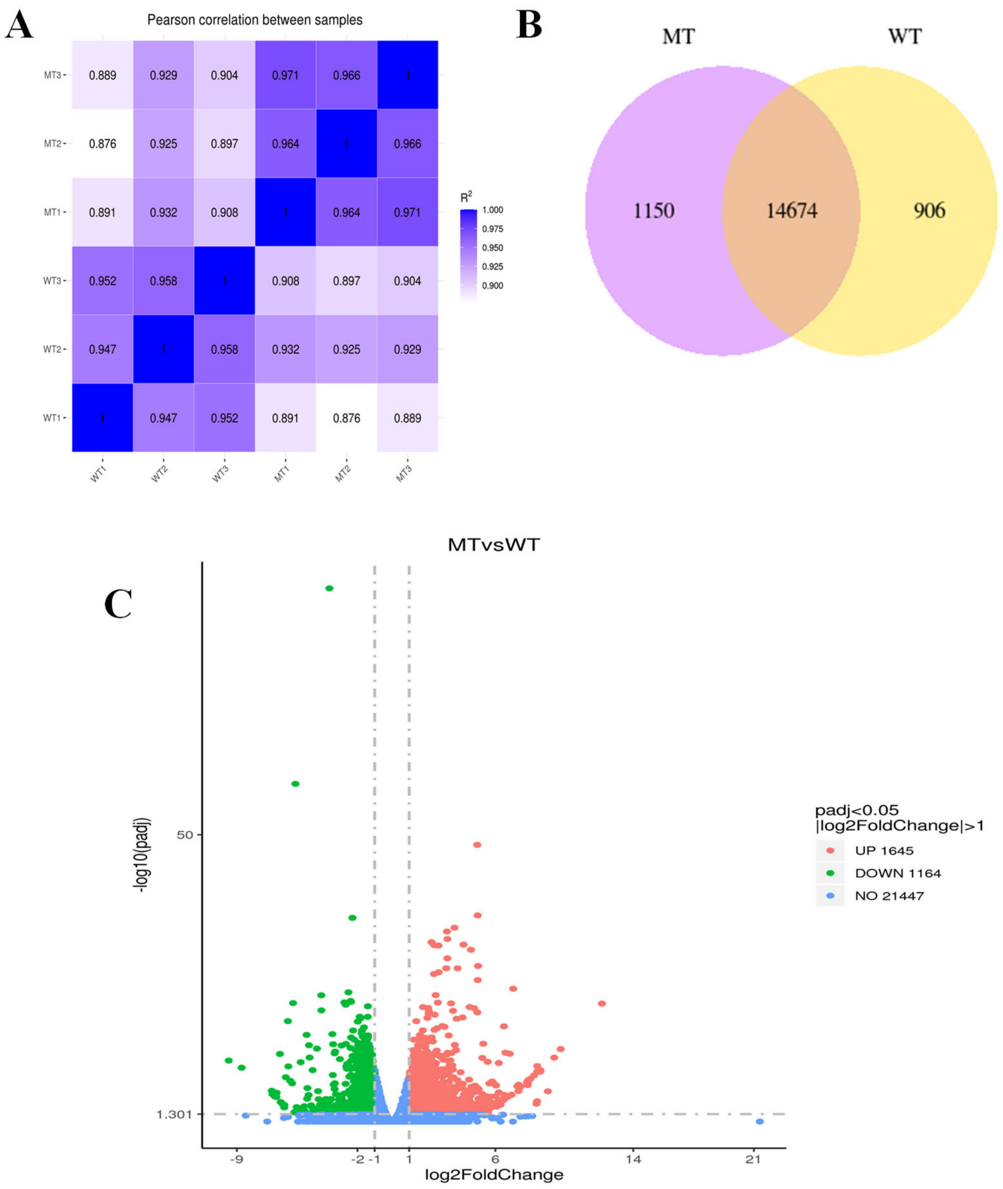

Supplementary video S – The video of sperm motility of WT and *dmrt3a*$^{-/-}$.

Video S1: The WT sperm video. **Video S2**: the *dmrt3a* mutant sperm video. See the video submitted on the webpage.

The following is the raw images involved in the manuscript:

Figure 2 Expression of *dmrt3a*

(B)

a

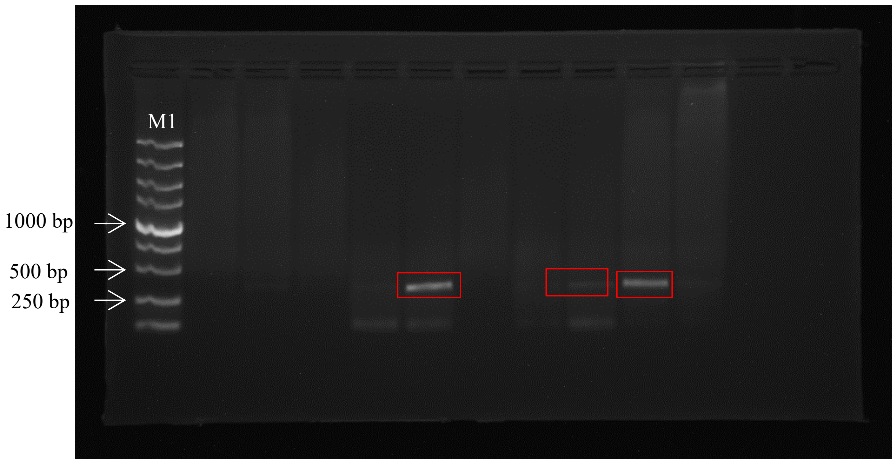

β-actin internal control

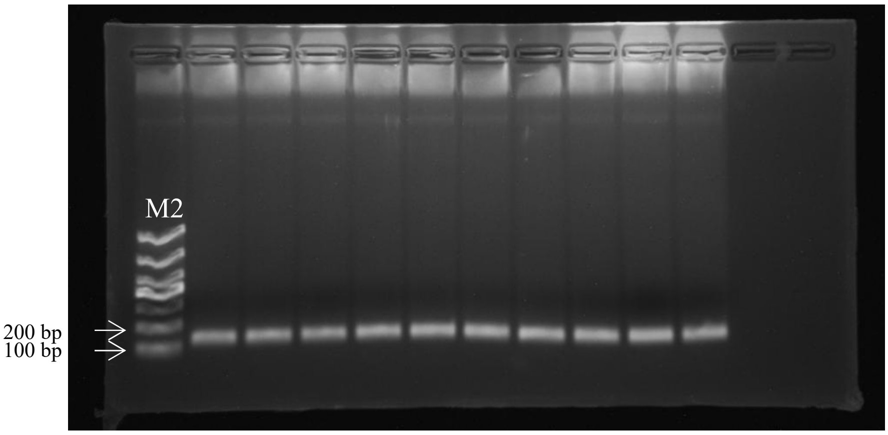

Supplementary Figure S2 – The process of screening and identifying mutants using PCR and T7 Endonuclease I.

PCR product

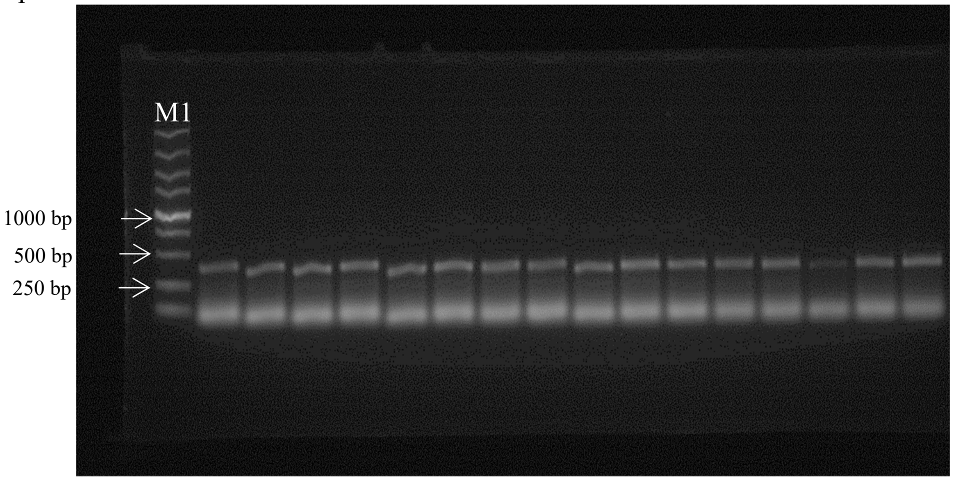

T7E1 digest PCR product

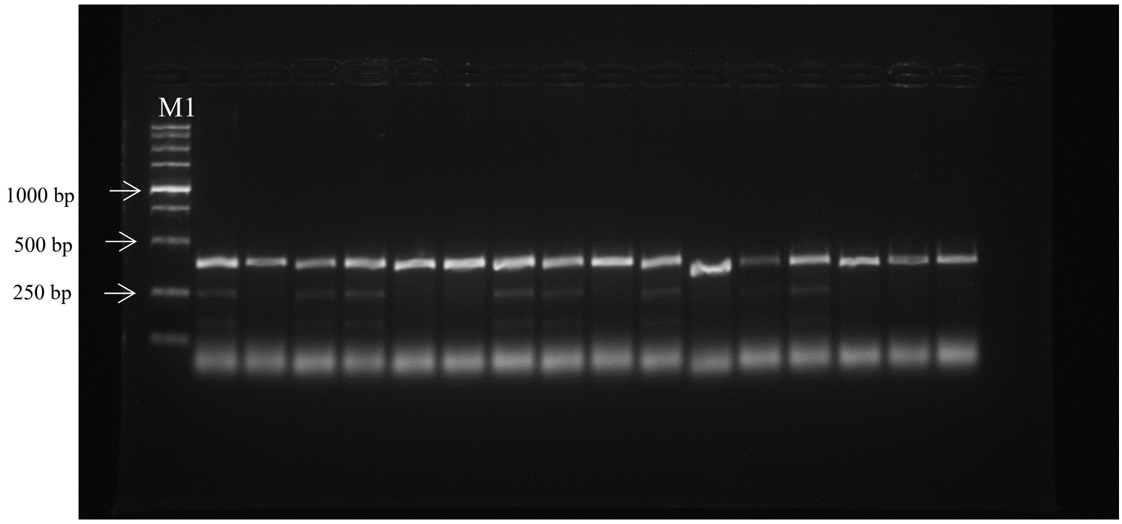

T7E1 digest PCR product（*dmrt3a*$^{-/-}$/WT）

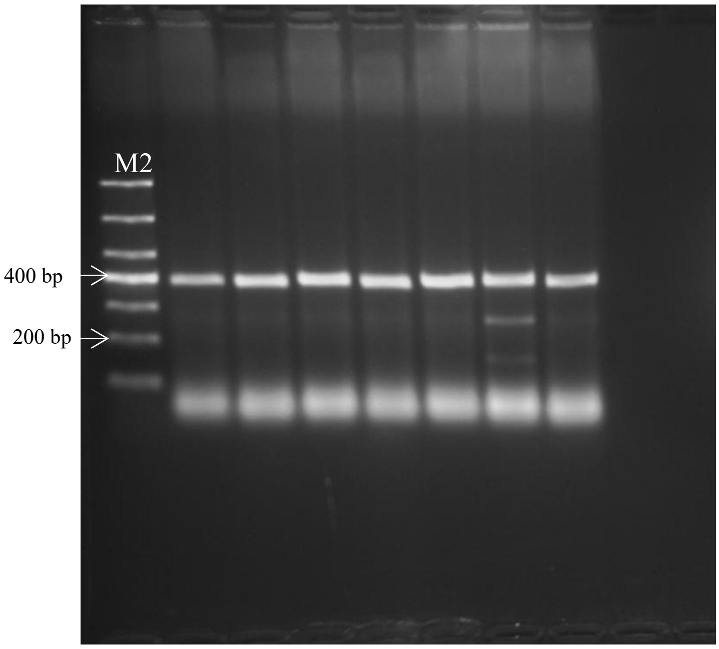

Supplement: Supplementary file 1 [file animals-14-02406-s001.zip › animals-3143447-supplementary/animals-3143447-supplementary/Supplemental- files- Tables and figures-dmrt3/Supplementary figures-dmrt3.pdf]
